# Effects of Synchronous Electrode Pulses on Neural Recruitment During Multichannel Microstimulation

**DOI:** 10.1038/s41598-018-31247-2

**Published:** 2018-08-30

**Authors:** James A. Hokanson, Robert A. Gaunt, Douglas J. Weber

**Affiliations:** 1 Department of Biomedical Engineering, Duke University, Durham, NC 27708 USA; 2 Department of Physical Medicine and Rehabilitation, University of Pittsburgh, Pittsburgh, PA 15213 USA; 3 Department of Bioengineering, University of Pittsburgh, Pittsburgh, PA 15213 USA; 4 Center for the Neural Basis of Cognition, University of Pittsburgh, Pittsburgh, PA 15213 USA

## Abstract

The recent proliferation of high-density microelectrode arrays has inspired several new applications of electrical microstimulation, including restoration of sensory functions in the visual, auditory, and somatosensory systems. In each case, the goal is to achieve precisely targeted activation of neurons, while patterning the location and timing of stimulation across the array to mimic naturalistic patterns of neural activity. However, when two or more electrodes deliver stimulation pulses at the same time, the electric fields created by each electrode interact. The effects of field interactions on neuronal recruitment depend on several factors, which have been studied extensively at the macro-scale but have been overlooked in the case of high density arrays. Here, we report that field interactions can significantly affect neural recruitment, even with low amplitude stimulation. We created a computational model of peripheral nerve axons to estimate stimulation parameters sufficient to generate neural recruitment during synchronous and asynchronous stimulation on two microelectrodes located within the peripheral nerve. Across a range of stimulus amplitudes, the model predicted that synchronous stimulation on adjacent electrodes (400 µm separation), would recruit 2–3 times more neurons than during asynchronous stimulation. Our results suggest that field interactions should not be ignored when designing multichannel microstimulation paradigms, even at threshold-level stimulus amplitudes.

## Introduction

Electrical microstimulation using microelectrode arrays is being investigated as a technique to modulate neural activity with a range of potential applications including restoration of somatosensation^1,2^, vision^3^, hearing^4^ and motor functions^5^. Many of these arrays penetrate neural tissue to be in close proximity to stimulation targets (neurons) in nerves, the spinal cord, and brain^6–8^. This proximity may allow for an improved ability to activate a specific subset of neurons as compared to non-penetrating arrays. Stimulation thresholds for electrodes that penetrate neural tissue can be reduced by factors of 10 to 100 over non-penetrating electrodes^6^.

Many stimulation paradigms using tissue-penetrating microelectrode arrays treat each electrode as an independent current source^5,9^, and microstimulation pulses are considered to influence recruitment only in a small volume surrounding the electrode^10^. Without scheduling to control the relative timing of pulses on multiple electrodes, stimulation paradigms may deliver pulses at the same time (synchronously) on two or more electrodes. Depending upon the stimulus parameters, tissue properties, and the distance between electrodes, electric field interactions during synchronous stimulation could modify neural recruitment and result in undesirable functional outcomes. In the case of macroelectrode arrays, these electric field interactions are known to have significant effects^11,12^. If these interaction effects persist at the microelectrode scale, it may become difficult to achieve desired functionality^13,14^.

In the field of cochlear implants, it has been known for many years that electric field interactions among neighboring channels must be considered when creating stimulation patterns. One of the earliest stimulation paradigms that addressed this is known as continuous-interleaved-stimulation^15^. In this method, stimulation pulses on neighboring channels are interleaved to avoid synchronous stimulation. This paradigm is thought to improve performance by providing a more reliable mapping between individual electrodes and the percepts they elicit^15^. However, cochlear implants have also been designed to exploit these electric field interactions to target recruitment of neurons between electrodes, creating so-called *virtual* channels, which have been shown to improve speech perception^16^. Similarly, nerve cuffs placed on peripheral nerves to elicit muscle contractions have also leveraged electric field interactions to target different compartments of the nerve^17,18^.

In contrast to the electrodes described above, microelectrodes that penetrate neural tissue are often within microns of excitable tissue resulting in recruitment thresholds approximately 2–3 orders of magnitude lower. At such low stimulus amplitudes, it is unclear whether electric field interactions will play a significant role in neural recruitment. Knowing the answer to this question is important, as applications such as restoring somatosensory feedback or vision might require recruitment of just a few neurons to evoke more naturalistic percepts^19^. If synchronous stimulation alters the recruitment patterns at the small amplitudes associated with microstimulation, the potential benefits of microelectrode stimulation could be reduced.

Even if a stimulation paradigm explicitly avoids synchronous stimulation on neighboring channels, it may be impossible to avoid synchronous stimulation across an array as the density and number of electrodes in arrays increases^20^. Therefore, a better understanding of how synchronous stimulation pulses interact at the microelectrode array scale will enable improved stimulus paradigms designed to either avoid or take advantage of these interactions.

Rather than focus on methods to improve selectivity, which have been studied extensively^11,21–32^, the primary aim of this paper is to compare patterns of neural recruitment around a pair of tissue-penetrating microelectrodes when pulses are delivered synchronously versus asynchronously. Our working hypothesis is that synchronous pulses give rise to nonlinear recruitment, activating more neurons than the simple sum of those recruited when pulses are delivered asynchronously by the individual electrodes. Although it is obvious that the electric fields generated by each pulse will interact linearly, the effects of these interactions on neural recruitment are unclear and have not been examined under parameter values used commonly for microstimulation. In order to address these issues, we used a common model of peripheral nerve axons^33^ to simulate the effects of electric field interactions on the number of axons recruited in a peripheral nerve. Stimulation from non-penetrating electrodes (e.g. for *in-vitro* cell cultures), as well as stimulation of cell bodies, are not modeled. We also briefly consider these results from the perspective of trying to leverage these interactions to enhance neural recruitment in a targeted way.

## Results

**Figure 1:**
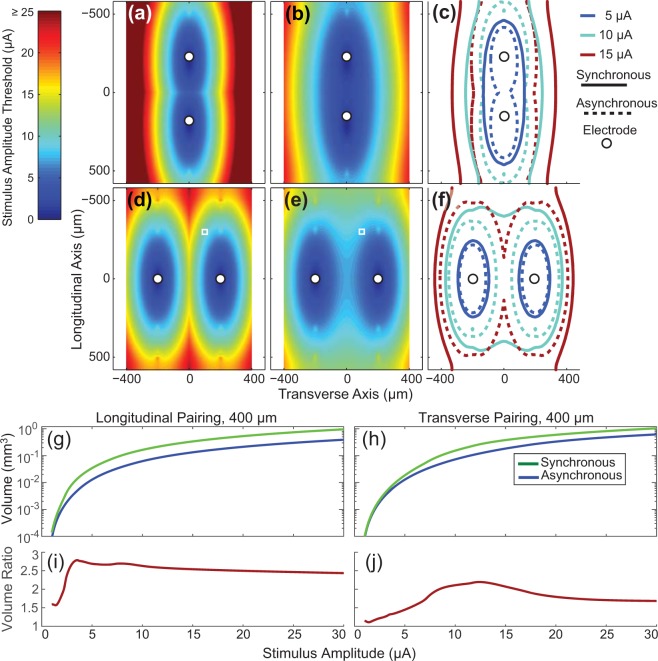
Computing the volume ratio. For ease of display, threshold results are shown in two dimensions although all numerical results are from computations done in all three-dimensions. Results for (**a**,**b**), (**d**,**e**) in this figure were computed using a 10 μm diameter fiber. (**a**) At each location the stimulus threshold of the axon in response to a single electrode stimulus is shown (asynchronous stimulation case). For (**a**) the electrodes are separated by 400 μm along the longitudinal axis (longitudinal pairing). (**b**) Similar to (A) except the threshold at each location is for synchronous stimulation. (**c**) Iso-threshold contours at 5, 10, and 15 μA for both the asynchronous and synchronous stimulation conditions. The solid lines represent the contours for synchronous stimulation and the dotted lines represent contours for asynchronous stimulation. (**d**–**f**), are the same as (**a**,**b**), and (**c**) except that they are for a transverse pair of electrodes spaced 400 μm apart. Recruitment volumes are shown in (**g**,**h**) for the longitudinal and transverse pairing, respectively. The ratio of recruitment volumes from synchronous stimulation over asynchronous stimulation produces the output variable of interest, the volume ratio (**i**,**j**). The small circles in panels a-f represent the location of the electrodes. The small white square in panels (**d**,**e**) indicate the spatial location of the center-most node of Ranvier from an axon relative to the electrodes as shown in Fig. 8, and shows how at this location, the threshold value is decreased when both electrodes are stimulating synchronously. Stimulus thresholds above 25 μA in (a) are saturated on the color scale shown to provide better visual detail for other other panels.

The change in peripheral nerve axon recruitment that occurs with synchronized stimulation pulses is shown in Fig. 1 for a 10 μm diameter fiber (1175 µm internodal distance) with two different electrode pairings. Figure 1a shows the thresholds for an axon positioned at different locations relative to two electrodes stimulating asynchronously. The threshold at each location is the lower of the two stimulus thresholds from the two electrodes and the colors illustrate the minimum stimulus amplitude required to recruit a neuron with a node of Ranvier at that location. Figure 1b shows the recruitment threshold at each point when the electrodes are stimulated synchronously. Although Fig. 1a,b demonstrate results at only a single tissue depth, axon recruitment at multiple tissue depths have been calculated, consistent with the idea of volume recruitment from penetrating electrode arrays. Synchronous stimulation decreased the threshold at all locations (3–50% reductions in threshold, median reduction 44%). Figure 1c shows the iso-threshold lines from Fig. 1a,b at 5, 10, and 15 μA to illustrate the growth in the volume of tissue recruited due to synchronous stimulation. This process is repeated in Fig. 1d–f for a transverse pairing of electrodes, which reveals 0–50% reductions in threshold (median 27%). The volumes of tissue activated (VTAs – see methods for more details) for the different stimulus conditions and electrode configurations are shown in Fig. 1g,h. The ratio of these recruitment profiles produces the volume ratio profiles shown in Fig. 1i,j. These results demonstrate that at low stimulation amplitudes the model predicts that synchronous stimulation will lead to a sizeable increase in the volume of tissue activated compared to if the electrodes stimulate at different times.

### Volume ratio and neuron ratio equivalence

**Figure 2:**
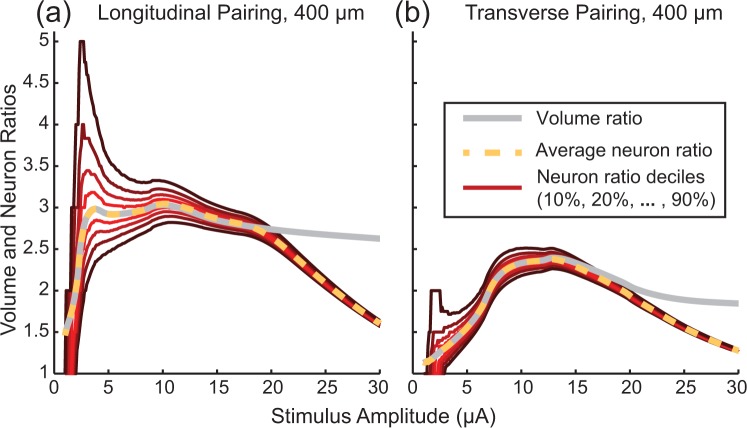
Comparison of Neuron and Volume ratios. Variability in neuron recruitment ratios for randomly placed populations of 14 µm diameter fibers. (**a**) Ratio of neurons recruited for randomly placed populations of 14 µm diameter fibers compared to the volume ratio using a pair of electrodes spaced 400 μm apart along the longitudinal axis. (**b**) Same as (**a**) but with electrodes spaced 400 μm apart along the transverse axis. Red lines indicate deciles of observed neuron ratios (number of neurons recruited during synchronous stimulation compared to the number of neurons recruited during asynchronous stimulation) starting at 10% and going to 90%. The average neuron ratio (50%) is shown as a dotted yellow line. The gray lines show simulation results for the volume ratios.

We were interested in comparing how the geometric volume ratio method used in this study compared to the ratio of neurons recruited using a more typical modeling approach where neurons are placed randomly in a volume. Here, the neuron ratio refers to the number of neurons recruited during synchronous stimulation relative to the number of neurons recruited during asynchronous stimulation. Each population consisted of 14 µm diameter axons randomly placed in an 800 × 800 × 1400 µm volume. Figure 2 shows the difference between the volume ratio and the recruited neuron ratio, computed for 10000 different random populations of 2038 neurons. At each stimulus amplitude, the resulting neuron ratios were broken down into deciles. At low stimulus amplitudes, inhomogeneities in the neuron populations drive large variation in the neuron ratio. The mean neuron ratio matches the volume ratio as expected. As the stimulation amplitude increases, the variability in neuron ratios decreases. At sufficiently high amplitudes (approximately 15–20 μA in these examples), every neuron in the population is recruited during synchronous stimulation, causing a reduction in the neuron ratios and a deviation from the volume ratio, which was not limited in spatial extent. The remaining results will be presented in terms of the volume ratio.

### Effect of fiber diameter

**Figure 3:**
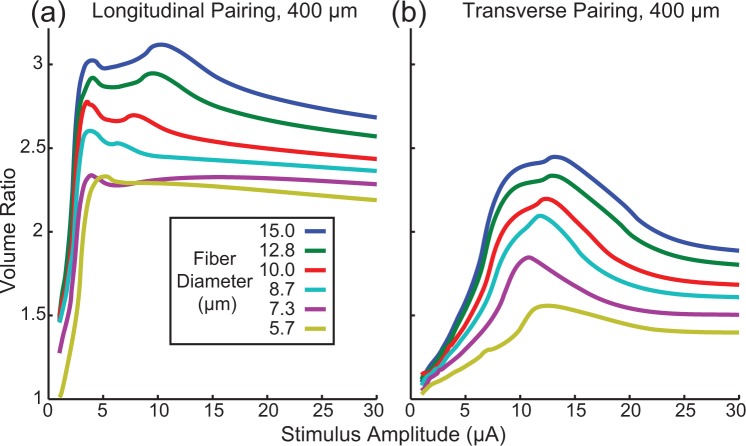
Volume ratios for different fiber diameters. Volume ratios for electrodes spaced 400 μm apart longitudinally (**a**) and transversely (**b**) for different fiber diameters. Axons with larger diameters had larger volume ratios than smaller diameter axons indicating the larger diameter axons are more susceptible to electric field interaction effects created when stimulation pulses occur synchronously on a pair of electrodes.

Peripheral nerves typically contain axons with a wide range of diameters^34^. Although experimentally we cannot manipulate these fiber diameters, it is important to understand how synchronous and asynchronous stimulation recruit axons with different diameters. Figure 3 shows how the volume ratio changes as a function of fiber diameter.

As was the case for the 10 μm diameter fiber, synchronous stimulation recruits more neurons than asynchronous stimulation for all fiber diameters tested (volume ratio always greater than 1). Note that a volume ratio of 1 indicates functionally independent electrodes, whereby synchronous stimulation recruits the same volume of tissue as asynchronous stimulation. A volume ratio of 2 indicates that synchronous stimulation recruits twice as much tissue as the sum (union) of the VTAs for asynchronous stimulation. We found that larger diameter neurons are more susceptible to these interaction effects for all stimulus amplitudes tested. Additionally, longitudinal electrode pairings have larger volume ratios than transverse electrode pairings.

### Effect of pulse width

In addition to changing stimulus amplitude, it is also common to vary stimulus pulse widths in neural stimulation experiments. In this context we are assuming that a particular pulse width would be chosen and that the stimulation amplitude would be adjusted to achieve the desired neural activation. In Figs 2 and 3, volume ratios were plotted as a function of stimulus amplitude. However, when varying pulse width, the “strength” of stimulation is not the same for different pulses widths at a given amplitude, as wider pulse widths recruit more neural tissue than narrower pulse widths^30^. Additionally, normalizing to charge per phase does not normalize stimulation strength, as at a given charge per phase, narrower pulse widths will recruit more neural tissue than wider pulse widths^35^. A comparison of volume ratios versus pulse widths should thus be done as a function of desired level of neural activation (i.e. at a particular number of neurons being activated, or similarly, a particular volume of tissue being activated from asynchronous stimulation). Thus, to examine the effect of pulse width, we chose to examine how the volume ratio changed as a function of the volume recruited during asynchronous stimulation. In other words, given a stimulus strength (combination of stimulation amplitude and pulse width) that is sufficient to recruit a desired amount of neural tissue during asynchronous stimulation, we wanted to know how synchronous stimulation would change the amount of neural tissue recruited, based on the selected pulse width.

**Figure 4:**
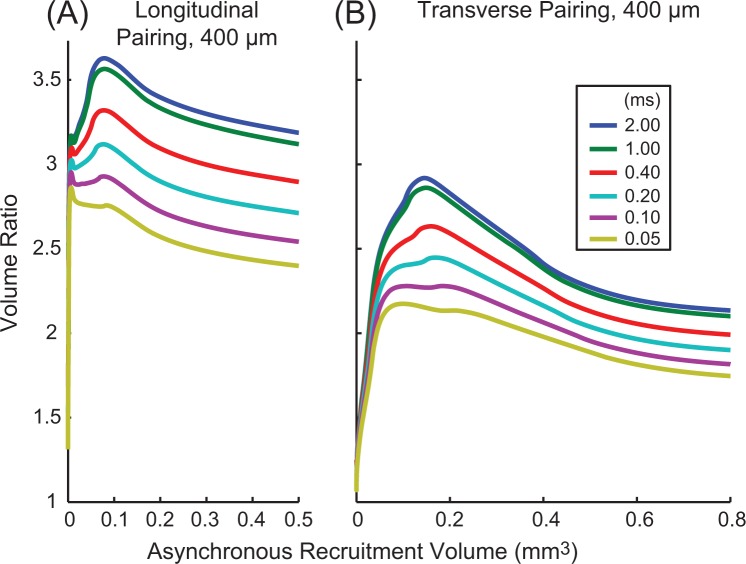
Volume ratios for different stimulus pulse widths. Volume ratios for electrodes spaced 400 μm apart longitudinally (**A**) and transversely (**B**) for different pulse widths. Results shown are for a 15 μm diameter fiber. Ratios are plotted as a function of the volume of tissue recruited during asynchronous stimulation, our measure of stimulation strength/effectiveness, rather than amplitude or charge per phase. Longer pulse-widths had larger changes in recruitment volume during synchronous stimulation.

Figure 4 shows the results of varying stimulus pulse width and amplitude for a 15 μm diameter fiber for longitudinal (Fig. 4A) and transverse (Fig. 4B) electrode pairings, plotted as a function of asynchronous stimulation recruitment volume, our proxy for stimulation strength/effectiveness. Wider pulse widths had higher volume ratios than narrower pulse widths. Only at the smallest recruitment volumes were the differences between stimulus pulse widths negligible. This trend was also true for smaller diameter fibers (not shown), although differences in volume ratio as a function of pulse width were smaller for smaller diameter fibers. Overall, these results suggest that narrower pulse widths would reduce the impact of synchronous stimulation.

### Dependence on electrode pairing distance

**Figure 5:**
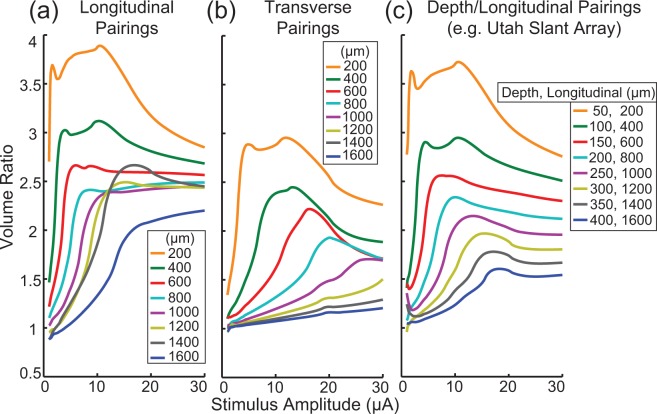
Volume ratios for differing electrode spacing between pairs of electrodes. Volume ratios for different spacings between pairs of electrodes separated along a longitudinal axis (**a**) and transverse axis (**b**) for a 15 µm diameter fiber (1450 µm internodal length). (**c**) Volume ratios as a function of electrode pairs which differ along both the longitudinal and transverse axes simultaneously (Utah Slant Array). In general, decreasing the spacing between the pair of electrodes increased the impact of synchronous stimulation.

To reduce the effect of stimulus interactions, it is possible to increase the distance between synchronously stimulated electrodes. Figure 5 shows the effect of stimulus interactions as a function of the distance between pairs of electrodes over a range of stimulus amplitudes. As expected, we found that electrodes that are closer together have larger volume ratios. Also, consistent with previous results, electrodes that are paired longitudinally exhibit larger ratios than transverse pairings. However, unlike the data presented in Figs 3 and 4, the change in the volume ratio as a function of stimulus amplitude for the longitudinal pairing does not show a smooth transition across the parameter being varied. This change, which is particularly noticeable at 1400 μm, is due to the relationship between electrode spacing and the internodal length of the axon. If electrodes are separated exactly by the internodal length of the axon, then each electrode will recruit the same group of neurons during asynchronous stimulation. In general, this will lead to an increase in the volume ratio (smaller denominator). The redundancy of neural activation from purely longitudinal pairings has motivated the development of arrays which covary along the longitudinal and depth axes, such as the Utah Slant Electrode Array^6^. This type of pairing is shown in Fig. 5c. Finally, for the longitudinal pairings, the larger electrode spacings (1200, 1400, and 1600 μm) appear to have volume ratios that are less than one at low stimulus amplitudes, although this effect is minimal and may be due to the employed 0.1 μA threshold accuracy.

### Sensitivity analysis for resistivity parameters

**Figure 6:**
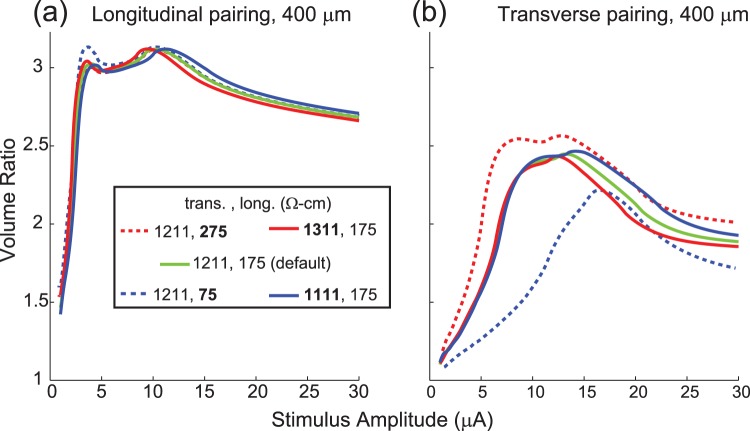
Impact of changing tissue resistivity on volume ratio. The tissue resistivity was changed to assess the impact of the resistivity on the volume ratio. Results shown are for a 15 μm diameter fiber for electrodes spaced 400 μm apart longitudinally or transversely. Resistivity values that have been changed from the default values are bolded in the legend.

We tested different resistivity parameters and found that they had little impact on the volume ratios for the longitudinal electrode pairings (Fig. 6a). For the transverse pairing (Fig. 6b), decreasing the longitudinal resistivity decreased the volume ratios while increasing the longitudinal resistivity increased the volume ratios. Changing the transverse resistivity had an opposite effect, although the effect was much smaller and only occurred at higher stimulus amplitudes. Similar results were seen for the other fiber diameters.

## Discussion

The key finding of this work is that even at very low amplitudes, synchronous microstimulation through multiple electrodes can have a large nonlinear effect on the recruitment of axons in peripheral nerves. This is especially important in the context of a high-density microelectrode array, where tens to hundreds of channels may be used and the probability of synchronous stimulation becomes high. These modeling results also suggest that field interactions among electrodes will be most significant for the large diameter axons in a given population (Fig. 3). Thus, taking into account the effects of synchronous stimulation is likely to be more important when targeting motor neurons with average diameters of 13–14 µm^34^ than, for example, the optic nerve where fiber diameters are on average only 1–2 um^36^. Narrower stimulus pulse widths will lessen the degree of interaction (Fig. 4). As expected, reducing the interelectrode spacing resulted in stronger interactions during synchronous stimulation (Fig. 5). These results were robust to changes in tissue resistivity (Fig. 6), an unknown parameter in the model.

In addition to this modeling study, several experimental studies lend evidence to there being behaviorally relevant interaction effects from synchronous microstimulation. In three separate studies, microstimulation pulses were delivered through neighboring electrode sites at different times, as well as synchronously, while differences in force recruitment were compared (Fig. 9a^6^, Fig. 3^22^, Fig. 3^27^). These examples include data that can be used to compute force ratios, which are similar to the volume ratios computed here in that they compare force recruitment from synchronous stimulation relative to the force data from each of the electrodes stimulating asynchronously. Due to a lack of detail in these reports, it is not possible to make direct comparisons to the volume ratio predictions made in the present study; however the force ratios (~3–30) are similar to, and likely even higher than the volume ratios observed in our model (Fig. 3, maximum peaks of ~1.5 to 3 across fiber diameters)

Given the effect of synchronous stimulation on neural recruitment, a stimulus paradigm designed on the assumption of electrode channel independence^9^ should avoid synchronous stimulation on multiple electrodes. Given high electrode counts in modern multielectrode arrays, it may not be possible to stimulate each electrode at a different time than all others, especially when high stimulus rates are used. Cochlear implants have partially solved this problem by narrowing the pulse width. In these systems, narrow pulse widths on the order of 40 µs or less are common^37^. Many cochlear implant stimulation paradigms also place each electrode in a group, where the group constituents are non-neighboring electrodes, to minimize interactions. Scheduling stimulation in this way is a useful approach for avoiding synchronous stimulation by allocating a specific time slot in which each stimulus channel can be active.

**Figure 7:**
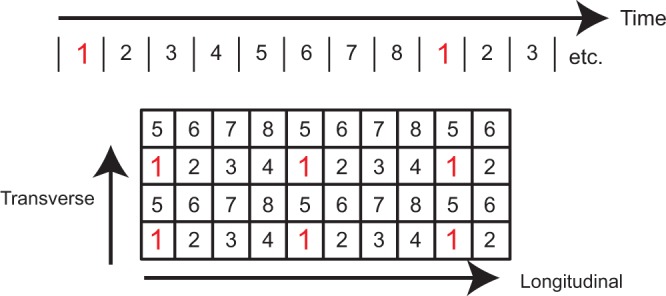
Illustration of stimulation scheduling for a two-dimensional electrode array. The large rectangle represents a 4 × 10 electrode array where each square represents an electrode channel. Each stimulus channel has been assigned to one of eight different time bins in which it can stimulate. Each electrode in a group is spatially separated from the other electrodes in the group to reduce the impact of synchronous stimulation. This approach to grouping reduces neuronal recruitment from synchronous stimulation, while allowing for higher stimulation rates than would be possible if every single electrode stimulated at a different time. Groupings should be further apart along the longitudinal axis than the other axes as interaction effects are stronger along the longitudinal axis (see Fig. 5).

The approach used by cochlear implant stimuli can be expanded easily to two or three dimensions (Fig. 7). In this example, the numbers at each electrode site indicate their group ID and electrodes within a group are allowed to stimulate at the same time. However, electrodes in a specific group cannot stimulate during another group’s time. Given the higher volume ratios observed along the longitudinal axis it will be necessary to have more groups along this axis. This solution highlights the general approach but is not meant to be prescriptive.

Based on the results presented, it is difficult to prescribe an electrode spacing where interaction effects can be ignored. Two factors make this difficult. The first factor is the interpretation of the functional significance of the volume ratio magnitude. Although its interpretation is straightforward, the impact that a specific volume ratio may have on a physiological outcome is not. For example, if the volume ratio is 1.4, and 10 neurons are recruited by two different electrodes stimulating asynchronously, it is unclear if the functional outcome would be negatively impacted by recruiting 4 additional neurons from synchronous stimulation. The second factor is the variability in the ratio as a function of the actual neuronal population surrounding the tissue. Although the results from Fig. 2 show relatively little variability with different random populations, assumptions made about the density and distribution of neurons might be inaccurate, and larger variability is certainly possible.

In summary, a scheduling approach can be used to guarantee that nearby electrodes are never stimulated at the same time. Although the ideal scheduling for maximizing electrode independence is to never stimulate electrodes at the same time, this may not be feasible. In that case electrodes should be grouped to stimulate together such that members of a group are far apart. Importantly, the distance metric is not physical distance but electrical distance. For peripheral nerves with high tissue anisotropy, electrodes along the longitudinal axis are electrically closer than those along the other axes, so electrodes in a group need to be further apart along the longitudinal axis (see Fig. 5).

Thus far we have considered interactions with the goal of maximizing electrode channel independence, assuming that recruitment of additional neurons may be undesirable. However stimulus interactions due to synchronous stimulation can be either detrimental or beneficial, depending upon the goals of a particular application. It is possible to design stimulation paradigms that take advantage of these field interactions, as is the case with current steering or focusing. This technique can be used to activate tissue that cannot be stimulated using a single electrode. For example, stimulation paradigms in clinical use for cochlear implants are starting to take advantage of stimulus interactions to improve performance^16^.

With penetrating microelectrode arrays, the proximity of the electrodes to tissue has largely led to an avoidance of the topic of field interactions, since the electrode density is relatively high, and stimulus amplitudes are relatively low. Additionally, in the field of motor stimulation, the technique of interleaving stimulation to avoid fatigue has led to an avoidance of the issue of stimulus interactions with penetrating arrays^38^.

The increased efficacy of recruitment during synchronous stimulation that we observed in the model, particularly in recruiting neural tissue between the electrodes, could serve as a current steering mechanism (see Fig. 1c,f for examples). In one example, Branner *et al*. reported that asynchronous stimulation through two different electrodes each maximally generated a muscle force of 4N^6^. However, when stimulated synchronously at amplitudes that generated 1 N each, a total muscle force of 6 N was generated; no explanation for exceeding a 4 N maximum was presented.

If we assume that the 4 N generated from a single electrode was sufficient to activate all muscle agonists, it is not immediately clear how the two electrodes together could generate 6 N. However, this observation makes sense if we consider co-recruitment of antagonists, which would reduce the observed force. In other words, maximizing muscle force requires recruiting all agonists and no antagonists, as any recruitment of antagonists will reduce the force generated. Thus, it is likely that by stimulating at lower amplitudes (those generating 1 N in isolation), synchronous stimulation was able to recruit more agonists and less antagonists, leading to the force increase. The 3x increase in force (6 N) from synchronous stimulation, relative to the sum of the forces generated from asynchronous stimulation (2 N), suggests a volume ratio of 3. This value is slightly higher than the maximum predicted from a model based on the reported spacing (2.1 for 14 μm diameter fibers from electrodes separated by 400, 200 and 800 μm in the transverse, depth, and longitudinal axes respectively, see also Fig. 5c).

Use of multiple electrodes is now starting to be studied as a method of increasing force recruitment^39^. In contrast to the more common notion of anodal inhibition to provide current steering^17,40^, it is synchronous cathodic stimulation, as was studied in this model, which is being used clinically for current steering in cochlear implants^16^. Recent modeling work has also suggested that this type of stimulation would be more advantageous than tripolar stimulation with guarding anodes^41^.

One limitation of this model was that the fascicular perineurium was not modeled. The fascicular perineurium acts as a high impedance barrier to charge^42^. It is expected that the perineurium would reduce the impact of synchronous stimulation for electrodes positioned inside different fascicles as they are electrically far apart^43^. However, for electrodes that are within the same fascicle it is expected that synchronous stimulation would become a more important factor, as charge is restricted within the fascicle. This may be one of the reasons why previous experimental data using muscle force to examine the effect of synchronous stimulation has shown even higher ratios than were seen with this model.

In summary, results from this study suggest that it is important to take into account the relative stimulus timing between stimulus channels when designing stimulation paradigms for high-density microelectrode arrays. Specifically, synchronous stimulation on adjacent electrode channels can cause large increases in the number of recruited neurons in comparison to asynchronous stimulation. This effect is increased for larger diameter fibers, wider stimulus widths, and closer electrodes. To maximize electrode independence, it is recommended that a stimulation scheduling approach is used where neighboring electrodes are only able to stimulate at non-overlapping times.

## Methods

All code necessary to reproduce this paper has been published at: https://github.com/JimHokanson/Hokanson_sync_stim.

### Determining the response of a cell to microstimulation pulses

A two-step computational modeling approach was used to determine the activation of cells in response to a variety of stimulus conditions^44^. For a single cell and set of stimulus parameters, we first computed the voltage applied to the cell as a function of the current delivered by one or two stimulating electrodes. Second, we simulated the cell’s response to the applied voltage to determine whether the stimulus was sufficient to elicit an action potential.

Cells were modeled using the program NEURON version 7.2^45^. For this analysis, only the axons were modeled (peripheral nerve model) and consisted of 21 nodes of Ranvier and 20 internodal regions. The morphology and channel kinetics are from McIntyre *et al*.^33^ as implemented in Model DB #3810^46^. Simulations were executed with a fixed time step of 5 µs using the default integration method (backward Euler). Simulations were run for 0.4 ms after the end of the stimulus pulse, which was sufficient to allow all tested fiber diameters to propagate an action potential to the end of the model.

Biphasic stimuli with a cathodic phase (default 200 μs) followed immediately by an anodic phase of half amplitude and twice the duration were used unless stated otherwise; This waveform has been used experimentally in our lab^47^. Reported amplitudes, varying from 1–30 μA, correspond to the magnitude of the cathodic phase.

The electrodes were modeled as point sources^48^ and the tissue was modeled as an infinite, homogenous, anisotropic medium with longitudinal resistivity of 175 Ω-cm (ρ_z_) and a transverse resistivity of 1211 Ω-cm (ρ_x_ and ρ_y_)^49,50^. The electric field from a single electrode was computed using equation (1)^51,52^.

1 $$V(x,y,z)=\frac{10\ast \sqrt{{\rho }_{x}{\rho }_{y}{\rho }_{z}}\,{I}_{stim}}{4\pi \sqrt{{\rho }_{x}{({\rm{\Delta }}x)}^{2}+{\rho }_{y}{({\rm{\Delta }}y)}^{2}+{\rho }_{z}{({\rm{\Delta }}z)}^{2}}}$$

**Figure 8:**
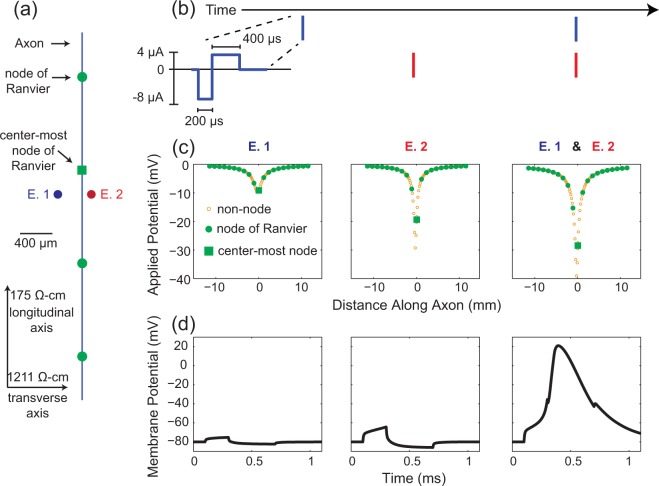
Synchronous versus non-synchronous stimulation of a single cell. (**a**) Spatial layout of the model system. Axons run parallel to the longitudinal (z-) axis. A 10 μm diameter fiber is shown with nodes of Ranvier spaced regularly at 1150 μm intervals. In this example, the center node of Ranvier is located 300 μm away from the electrodes along the longitudinal axis, and 300 μm and 100 μm away from electrodes E. 1 and E. 2, respectively along the transverse axis, and at the same depth as the electrodes. The electrodes are thus separated by 400 μm along the transverse-axis (transverse pairing). (**b**) Diagram of the stimulus-waveform as well as indicating the relative timing to stimulus pulses on both electrodes. For (**c**,**d**) the left and middle panels correspond to stimulation on electrodes E. 1 (left) and E. 2 (middle) asynchronously. The right panels correspond to synchronous stimulation on both electrodes. (**c**) The potentials applied to the axon model from the cathodic phase of the stimulus waveform. The potentials from the anodic phase would have opposite polarities and half the amplitude. Potentials are computed using eqn (1). (**d**) Membrane potential at the centermost node of Ranvier during the simulation as computed using NEURON. This example demonstrates that although neither electrode is able to activate this axon when stimulated individually, synchronous stimulation on both electrodes elicits an action potential.

V (mV) indicates the voltage at a point some distance away from the stimulating electrode relative to a distant ground. I_stim_ (µA) represents the stimulation current amplitude. ∆*x*, ∆*y*, and ∆*z* are computed as the distance (in µm) between each electrode and the point along an axon where V is being computed. A scaling factor of 10 is included to account for unit conversion. For each phase of the stimulus, the discrete neural solver, NEURON, is updated with a new set of external voltages to apply from equation (1). The electric field generated by multiple electrodes is computed as the sum of the fields from each individual electrode^44,53^ (principal of superposition). In all simulations, axons run parallel to the z-axis with the x, y, and z axes being referred to as the transverse, depth, and longitudinal directions, respectively. Figure 8a shows a portion of an axon oriented parallel to the longitudinal axis with a pair of electrodes (E.1 and E.2) positioned at the same longitudinal distance along the axon but at different transverse distances. Figure 8b shows the timing of biphasic current pulses delivered on the two electrodes. The resulting potentials generated by the cathodic phase of the stimulus pulses is shown in Fig. 8c. Although the stimulus waveform is the same for both electrodes, electrode, E.2 is closer to the axon and thus generates larger potentials along the axon. Figure 8d shows the membrane potential at the center most node of Ranvier during the simulation. In this case, when the pulses are delivered asynchronously by the 2 electrodes, the resulting change in membrane potential is insufficient to elicit an action potential. However, when both electrodes deliver stimulation pulses synchronously, the applied potential is sufficient to generate an action potential.

### Examining the population response: computing a threshold lookup table

It is common to simulate recruitment thresholds using a population of randomly positioned neurons^54,55^. Here, we computed the threshold stimulus amplitude for an axon with its center-most node of Ranvier located at a particular 3D location relative to the stimulating electrode. This process was repeated in a uniform 3D grid in steps of 20 µm. In preliminary testing, this grid resolution led to a threshold error of less than 0.1 µA on average for intermediate locations.

A threshold amplitude was reached when a propagating action potential exceeding a membrane potential of 10 mV at the last node of Ranvier occurred. Thresholds were computed to 0.1 μA accuracy. When two electrodes were stimulated synchronously, the stimulus amplitude of both electrodes was always equal.

### Volume of tissue activated

Using this methodology, we computed the volume of tissue activated (VTA) by stimulation on one more electrodes at a particular stimulus amplitude. The VTA^56,57^ is a more general term for the more commonly used “sphere of activation” and is preferable in cases in which the recruitment volume is non-spherical. Neurons that have at least one node of Ranvier within this volume will fire an action potential in response to the stimulus. Although the shape of this volume may be interesting in some cases^57^ our analysis primarily reports on the magnitude of the VTA.

We computed the VTA by upsampling the 3D threshold grid to 1 μm spacing using linear interpolation, then numerically integrating the threshold values. We limited the volume along the longitudinal axis to the internodal length of the fiber diameter being examined, beyond which additional axons are not recruited^56^.

### Computing the impact of synchronous stimulation

The VTA was computed for a set of electrodes with both synchronous and asynchronous stimulation conditions. Later in our analysis we relate this to the number of neurons recruited, which depends upon the density and number of axons in the volume. To examine the effect of stimulus timing, we computed the ratio of the VTA during synchronous stimulation to the VTA during asynchronous stimulation. The ratio of these two values, referred to as the volume ratio, is an indication of how much more neural tissue is recruited due to synchronous stimulation. The following is an example of the calculation of the volume ratio for a pair of electrodes.

2 $$Volume\,Ratio=\frac{Volum{e}_{synchronous}}{Volum{e}_{asynchronous}}=\frac{Volum{e}_{(1,2)}}{Volum{e}_{(1)}\cup Volum{e}_{(2)}}$$

Volume_(1,2)_ represents the volume of tissue activated by both electrodes stimulating synchronously whereas Volume_(1)_ and Volume_(2)_ represent the volumes of tissue activated by each electrode, stimulating asynchronously (non-overlapping pulses and without any neuronal hyper/de-polarization from previous stimuli). Here the symbol ‘∪’ indicates the union of the two volumes, i.e. the linear sum of VTAs for both electrodes, making sure not to double count tissue. When calculating the union along the longitudinal axis, additional care must be taken to not double count tissue which corresponds to activation of two different nodes of Ranvier from the same axon.

### Simulating randomly placed neurons to estimate number of neurons recruited

Neuron recruitment in this paper was tested with nodes-of-Ranvier placed at uniform locations in a 3D volume. This approach was used to facilitate threshold predictions based on previously solved thresholds for neurons in the grid. These predictions were used to limit the range of threshold values for testing, which reduced the number of simulations needed to find threshold using a binary search algorithm. Additionally, this approach required no estimate of neuron density or neural-extent.

In contrast, traditional methods of modeling neuron recruitment tend to compute population statistics on randomly placed neurons rather than using activation volumes. By randomly placing neurons, one can track the activation of a particular neuron under synchronous and asynchronous stimulation conditions. This result can be used to compute a neuron-ratio, comparing the number of neurons recruited during synchronous stimulation to the number of neurons recruited during asynchronous stimulation. We ran additional simulations using randomly placed neurons to compare the resulting neuron ratios to results obtained from the volume ratio (equation 2).

10000 different random populations of 14 µm diameter fibers were simulated. For each simulation, center-node locations were generated randomly from a uniform distribution, with the range of the locations limited to a 800 × 800 × 1400 µm (transverse, depth, and longitudinal axes respectively) volume around the stimulus electrodes. Each population consisted of 2038 axons. This choice of fiber diameter and density was based on Mahnam *et al*.^55^, which was chosen as a recent example of generating a random distribution of fibers of roughly fixed diameter (see also^58^). For each population, the number of neurons responding to synchronous and asynchronous stimuli was determined. The resulting neuron ratios were compared to the volume ratio.

### Effect of varying fiber diameter, pulse width, and electrode spacing

Using the volume ratio calculation (equation 2), we investigated the effects of changing the fiber diameter, stimulus pulse-width, and the spacing and orientation between a pair of electrodes. Fiber diameters were changed from 5.7 μm to 15 µm and used to explore the impact of synchronous stimulation on recruitment of different fiber diameter populations. Stimulus pulse-widths were varied between 50 µs and 2 ms. Finally, electrode spacing was varied between 200 µm and 1600 µm in increments of 200 µm along the longitudinal and transverse axes, and simultaneously along the depth and longitudinal axes in a manner meant to mimic the Utah Slant Electrode Array^6^. For each varied parameter, the impact of synchronous stimulation was assessed as a function of the varied parameter and stimulation amplitude.

In addition to the primary model manipulations, we conducted a sensitivity analysis on the resistivity parameter as we are unaware of published measures of mammalian peripheral nerve resistivity. Other similar modelling studies use values based on measurements of the dorsal columns of the spinal cord^49^. To examine the impact of the resistivity on our results, both longitudinal and transverse resistivities were changed by ±100 Ω-cm while holding the other constant.
